# The study of mindfulness as an intervening factor for enhanced psychological well-being in building the level of resilience

**DOI:** 10.3389/fpsyg.2022.1056834

**Published:** 2022-12-21

**Authors:** Vincent Kim Seng Oh, Abdullah Sarwar, Niaz Pervez

**Affiliations:** Faculty of Management, Multimedia University, Cyberjaya, Malaysia

**Keywords:** psychological well-being, mindfulness, adversity, resilience, millennials

## Abstract

**Background:**

By using a practice like mindfulness, people may become more adaptable and flexible in difficult situations, which lowers the levels of unfavorable experiences. Only a small number of research have examined the connection between mindfulness and resilience, with mindfulness as a source of PWB influencing millennials’ resilience when faced with adversity. This study sought to close this gap by exploring the role that mindfulness practice plays in millennials’ PWB and subsequent increases in resilience to adversity.

**Methods:**

In this study, key components linked to mindfulness, PWB, and resilience are combined with a thorough literature assessment. Millennials who are active members of the Ti-Ratana Youth in Malaysia and the Buddhist Missionary Society of Malaysia (BMSM) Youth Section make up the study’s sample population. Before completing the online survey form, each participant was instructed to engage in 4 weeks of supervised mindfulness practice. To assure the validity of the data gathered, it was crucial to secure the youth’s commitment. Only 231 of the 300 respondents who received the link to the online survey had replies that could be used for further research. To analyze the collected data and conduct hypothesis testing, Smart-PLS was used.

**Results:**

Academic research has shown that factors, such as a heavy workload, time constraints, lengthy workdays, work-related home conflicts, and an unstable and uncertain environment all lead to a drop in PWB. According to the findings, the two most significant variables promoting resilience are mindfulness and positive PWB. The outcomes of this experimental study confirmed earlier findings that millennials’ PWB and resilience are enhanced by mindfulness training.

**Conclusion:**

The study’s findings suggest that, in times of high uncertainty, mindfulness-based intervention programs should be expanded to include all young people. This study offers empirical support for the effectiveness of mindfulness-based interventions in raising PWB and resilience.

## Introduction

Resilient people are often optimistic, emotionally sensitive, socially sensitive, and able to face problems without losing perspective or the capacity to carry out daily chores (Babić et al., 2020). In other words, resilience is the ability to tackle obstacles head-on while remaining optimistic. Resilience, defined as the ability to remain buoyant, adjust flexibly, or perhaps grow in the face of pressures or adversity (Bogaerts et al., 2021), might be a protective element to help millennials in their daily employment. Many millennials lack the skills and resources necessary to thrive in today’s challenging workplace (Nabawanuka and Ekmekcioglu, 2022).

When facing a difficult time, it is deemed that employees will be able to adjust to the rapidly shifting conditions. For example, to illustrate the ability to adapt to the shifting environmental needs, the World Health Organization (WHO) announced that the COVID-19 virus reached the pandemic level on March 11, 2020 (Kondratowicz et al., 2021; Weigelt et al., 2021). The rapid and complete relocation of one’s life, both professionally and personally, to a more secluded location, was precipitated by concerns regarding the presence of an unidentified disease, as well as ambiguity, and governments in various countries have imposed movement’s restrictions to control the outbreak (Wang et al., 2020; Xiong et al., 2020). Due to the movement restrictions imposed by various governments worldwide to prevent the spread of the disease, these restrictions have affected approximately 68% of the workforce worldwide. Their respective nations imposed pandemic limitations [International Labour Organization (ILO), 2020]. This unforeseen circumstance resulted in a reduction of almost 71% in people’s levels of well-being (Kondratowicz et al., 2021). In their personal and professional life, people have experienced both positive and negative moments during these adverse situations. The existence of such uncertainty and adversity in the lives of humans is unavoidable.

The COVID-19 epidemic has a growing body of data suggesting it has a negative impact on the PWB of many people, according to Solomou and Constantinidou (2020) and Wang et al. (2020). An individual’s PWB is related to the level of resilience and mindfulness (Friedman et al., 2022). According to Tan et al. (2021), PWB is an individual’s capacity to maintain a healthy equilibrium among various ideas, feelings, and circumstances, solve problems, and react to uncertainty and adversity.

Resilience is a feature that many people possess. A process of adaptation is the ability to be resilient. Resilience is a learned pattern of behavior that one acquires over time; it is not the result of a personality attribute (Sabouripour et al., 2021). The second dependent variable is a psychological practice of deliberately focusing one’s attention on the present moment known as mindfulness. One thing about these two variables is the same: they both involve a process (Li, 2021). All of these are processes that can be learned through practice. For these processes to be strengthened, the deliberate practice had to be reinforced by lifelong, dynamic learning, and experience (Friedman et al., 2022).

In the face of difficulty, trauma, tragedy, discomfort, threat, and other causes of stress, resilience and mindfulness are the processes of adaptation. Millennials may choose to intentionally consider ways to approach this pandemic outbreak by taking a different perspective, re-evaluating their assessment, and considering how it will affect their own lives and the lives of others (Kim, 2022).

Mindfulness practice involves bringing one’s attention to the here and now while remaining non-reactive, non-judgmental, and open-hearted (Friedman et al., 2022). The levels of adverse experience can be reduced because of practicing mindfulness since it is a technique that enables them to be flexible and adaptive in challenging settings. Increased attention, awareness, flexibility (both cognitive and response), tolerance, and a decrease in preconception and misunderstanding are all benefits of practicing mindfulness. Because of this, employees can act professionally rather than react automatically (Linder and Mancini, 2021).

According to Ryff and Singer (2003), resilient people can preserve their physical and mental health and may recover more quickly from the effects of adverse experiences. In recent years, there has been increased interest in studying mindfulness theories and their relationship with PWB and resilience. As a result, this study investigates the relationship between mindfulness, PWB, and resilience. It was hypothesized that mindfulness might significantly correlate with PWB and resilience. Higher levels of mindfulness indicate higher PWB and greater resilience. In addition, it was anticipated that engaging in mindfulness activities would contribute considerably to an individual’s overall level of resilience.

However, limited studies study the relationship between mindfulness and resilience, with mindfulness as a source of PWB affecting millennials’ resilience when facing adversity. This paper aims to examine the relationship between mindfulness and PWB among millennials to be resilient when facing adversity.

## Literature review

### PWB

Psychological Well-Being (PWB) stands for all psychological elements that are thought to contribute to a person’s overall quality of life (Kim, 2022). According to Tang et al. (2019), PWB promotes life satisfaction, and positive effects, and decreases negative effects. As a result, efforts should be made to improve the PWB of millennial workers who are under stress at work. A variety of factors, including mental, psychological, and social factors at the individual and organizational levels, could be used to explain the factors affecting the PWB.

### Resilience

Resilience is the capacity to successfully adapt by acting flexibly in response to changing situational demands or by exercising appropriate self-control in a demanding setting. When faced with shifting settings, people with resilience can maintain or improve their equilibrium and respond flexibly to stressful situations (Shin, 2021). Additionally, it lessens the detrimental effects of risk factors on mental health, such as stress. By reassessing stress, improving their resilience to stress, regulating negative emotions, and harnessing good benefits, those with strong resilience can demonstrate a rapid recovery (Chang, 2021). As a result, millennial employees may be better able to deal with a variety of challenging environmental circumstances.

### PWB and resilience

Ryff and Singer (2003) found that resilient people could safeguard their physical and PWB and recover from challenging situations based on a review of previous literature. According to Acciari et al. (2019), PWB and resilience levels were intertwined. According to Ryff and Singer (2003), resilient people are those who can handle pressures while keeping their bodily and mental health.

### Mediating variable

#### Mindfulness

The state of increased awareness known as mindfulness is achieved by paying deliberate, non-judgmental attention to the present (Marenus et al., 2021). Exercise that combines kinesthesia and physical effort is known as mindful exercise (Mitchell, 2021). People who practice mindfulness appear more able to control unpleasant emotions and ideas, and they are better able to absorb and learn from their experiences without judgment (Kabat-Zinn, 1994; Sünbül and Güneri, 2019).

#### Mindfulness and PWB

According to the various systematic reviews and meta-analyses by these authors, Kong et al. (2015), Garland et al. (2017), McConville et al. (2017), Feller et al. (2018), Li (2021), Søvold et al. (2021), Tan et al. (2021), Mubarak et al. (2022), Su et al. (2022) and all these authors have suggested that therapies like mindfulness training may improve PWB. Similarly, it is mentioned that an enhanced level of mindfulness is shown to have a significant relationship with reducing depression and stress, leading to better self-control and a more positive outlook on life (Lo et al., 2018; Epel et al., 2019) Mindfulness-based training has been studied extensively for its potential to improve mental health and happiness. Mindfulness practices promote positive adaptation to adversity, which reduces psychological distress, raises happiness levels, and improves PWB (Jiga et al., 2018).

#### Mindfulness and resilience

The term resilience describes an individual’s capacity for dealing with adversity, whether that adversity originates internally or externally (Zsido et al., 2022). PWB was favorably correlated with resilience, whereas psychological discomfort (such as depressive and anxious symptoms) was adversely associated with resilience (Arslan, 2019; Kwok et al., 2020).

Based on the study by O'Connor et al. (2021), the level of mindfulness is a crucial resource for building individual resilience levels in the face of adversity. It is an essential resource because practicing mindfulness can encourage the development of several traits, such as emotional control, healthy coping mechanisms, and a healthy sense of self-worth, that serve individuals well when facing life challenges and the environment.

This practice may aid in realizing that the adverse events that lead to psychological distress are merely a natural mental reaction and temporary or short-term when faced with adversity. The realization may improve one’s ability to tolerate and positively cope with such adverse experiences. Multiple empirical studies have shown similar results that show a strong relationship between the constructs of mindfulness, greater life satisfaction, resilience, and significantly reduced levels of psychological distress in the general population, both adults and children (Hopkins et al., 2014; Van der Gucht et al., 2014; Bamishigbin et al., 2017; Lo et al., 2018).

With that, the effort to enhance resilience is gained through mindfulness practice and, in turn, will improve PWB. With that, the purpose of this study is to conduct a study among millennials and formulates the hypothesis that mindfulness practices are significantly correlated with the two constructs of PWB and resilience.

## Research model

**Figure 1:**
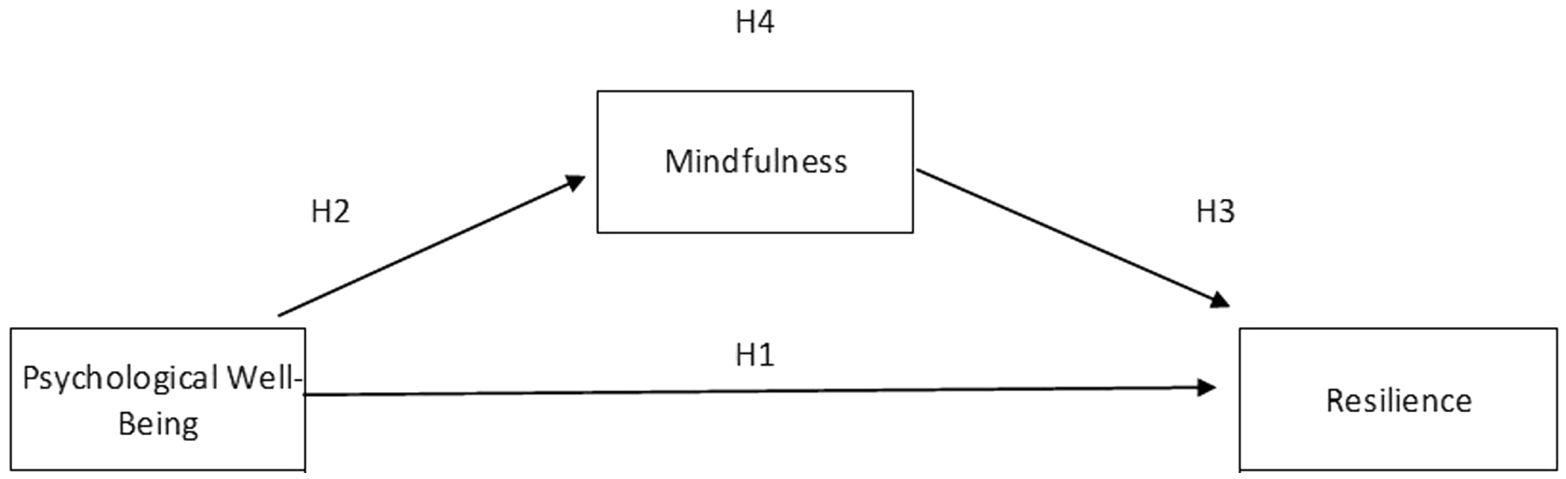
A research framework.

The study hypothesizes that practicing mindfulness can help improve a PWB. Those who engage in any mindfulness practice will experience increased levels of awareness and a significant increase in their resilience and PWB (Li, 2021). This positive change will enable one to better deal with uncertainty and adversity resulting from the challenging environment. The target population of this study is millennials, who may have to deal with various uncertain challenges from their work and family. Figure 1 presents the model that was used for the research.

The most common challenges are heavy job load and responsibilities, prolonged working hours, datelines pressure, the uncertainty of the changes in the business environment, and challenges related to a disruption in the work-life balance. The practice of mindfulness helps employees increase their awareness and improves their ability to observe and respond to situations more effectively (Friedman et al., 2022). This capacity allows employees to cope better in adverse conditions, improving their PWB.

## The hypothesis

The hypotheses are formulated based on research that suggests that practicing mindfulness significantly lowers people’s stress levels and raises their well-being and resilience.

*H1*: There is a significant correlation between PWB with resilience

*H2*: There is a significant correlation between PWB with mindfulness

*H3*: There is a significant correlation between mindfulness with resilience

*H4*: The relationship between PWB and resilience is significantly mediated by mindfulness, which considerably impacts both constructs.

## Methodology

A convenient sampling technique is used to collect the samples. Millennials who are active members of Ti-Ratana Youth and the Buddhist Missionary Society Malaysia (BMSM) Youth Section make up the samples. Both are local youth organizations with members in Malaysia that range in age from 18 to 40. The group’s active members, the majority of whom are employed, and their awareness of mindfulness make them suitable participants for our study. The reason for incorporating the youth organizations is that the study’s participants are required to engage in 4 weeks of supervised mindfulness practice. The young people had to daily engage in guided mindfulness exercises. Therefore, it is crucial to secure the youth’s commitment to assure the validity of the data gathered. The participants actively participate in the youth group, which facilitates easy contact and study monitoring, to handle the commitment concerns. The 4 weeks of practice began after participants provided their informed consent to take part in the study. All participants were requested to complete a questionnaire that was given online after the 4 weeks of practice.

A total of 300 questionnaires were sent to the target respondents who fit the profile for this study. After the initial screening for any missing and invalid responses, a total of 231 responses (*N* = 231) were found valid to test the proposed conceptual model. Smart-PLS were utilized to analyze the gathered data and hypothesis testing which includes validating the measurement and structural models and testing the proposed hypotheses. In this study, evaluating the measurement model was carried out by taking into account various factors, including convergent validity, discriminant validity, internal consistency reliability, and indicator reliability (Henseler et al., 2015; Hair et al., 2019). Measures of multicollinearity, path co-efficient, coefficient of determination (R2), effect size (f2), and predictive relevance (Q2) were utilized in the process of evaluating the structural model (Hair et al., 2017). Every one of these procedures was carried out precisely as described. This analysis employs PLS-SEM because it permits a simultaneous investigation of the constructs and the structural model upon which they are based. It is helpful in exploratory and survey research (Hair et al., 2019). According to Ringle et al. (2015), PLS-SEM is the method of choice for evaluating path diagrams for latent variables when many indicators are involved. This is because PLS-SEM may simultaneously analyze formative and reflective models while maintaining possible advantages over linear regression models.

## Measures

### Mindfulness

Mindful Attention Awareness Scale, often known as the MAAS, examines mindfulness as a characteristic that involves what its creators consider to be the two fundamental aspects of consciousness: awareness and attention. The MAAS measures the awareness of the level of attention to present-moment events and experiences. It contains 15 items for the respondents to rate based on a scale ranging from 1 (almost always) to 6 (almost never) based on the frequency with which the respondents engage in the described activities. In this particular research, the scale demonstrates a high degree of internal consistency (Cronbach’s α =0.906; refer to Table 1).

**Table 1:** Cronbach’s alpha, composite reliability, and AVE of the measures.

	Cronbach’s alpha	Composite reliability	Average variance extracted (AVE)
MF	0.906	0.919	0.651
PWB	0.854	0.880	0.589
RES	0.677	0.770	0.526

### PWB theory

A person’s PWB level can be evaluated using the Scale of PWB (SPWB), developed by Ryff (1989). Positive functioning is an alternative and multi-model of PWB that originates from theoretical talks on how normal personality development takes place. This served as the foundation for Ryff’s model, which he founded on this concept. The model provides a comprehensive explanation of what mental health entails. On the scale of PWB, there are a total of six elements, and each of those aspects is comprised of 18 items. It was devised to gage independence, environmental dominance, constructive connections with others, personal development, life objectives, and self-acceptance. A reverse scoring item is included in the list of questions to determine whether respondents are giving consistent answers. The respondents were to rate based on a scale ranging from 1 (strongly agree) to 7 (strongly disagree) based on the respondent’s opinion on their level of PWB. The internal consistency shows a high degree of the outcome (Cronbach’s α =0.854; refer to Table 1).

### Resilience

Using the CISS framework of the Coping Inventory for Stressful Situations to test the level of resilience and the ability to cope with stressful situations. The initial version of the instrument, which consists of 48 questions, was designed to gauge the various strategies used by participants to deal with stressful events in their life (Masten and Barnes, 2018). The CISS-SFC consists of 21 items measuring three aspects of coping: task-oriented, emotion-oriented, and avoidance-oriented. To identify which coping methods, the participants employ for various stressful situations. The participants must rate each item on a five-point Likert scale, with 1 (not at all) and 5 (very much). The internal consistency of this scale shows a moderate degree outcome (Cronbach’s α = 0.677; refer to Table 1).

## Results

### Preliminary analyses

Table 2 presents the demographic characteristics of the respondents recruited to provide their survey opinion for this study. The majority of the respondents, or 55.8%, are female, while the remaining proportion (44.2%) are male (*N* = 231 for male and female respondents combined). When broken down by age group, the most significant proportion of respondents fell into the 26–30 age bracket (34.7%), followed by the 36 and older age group (30.6%). Regarding the level of education, most respondents held a bachelor’s degree (54.2%). The following demographic is the occupation category, and most of the people who filled out the survey were employed in managerial positions (26.4%), followed by administrative functions (33.3%). Finally, regarding the number of years of service, the majority have been working for more than 5 years (40.3%).

**Table 2:** Demographic profile of the respondents.

	Frequency	Percentage		Frequency	Percentage
**Gender**					
Male	102	44.2			
Female	129	55.8			
Total	231				
**Age**			**Occupation**		
21–25 years old	29	12.5	Operational (work floor)	48	20.8
26–30 years old	80	34.7	Administrative	77	33.3
31–35 years old	51	22.2	Supervisor (Middle Management)	45	19.4
36 and above	71	30.6	Manager (Upper Management)	61	26.4
Total	231		Total	231	
**Education**			**Years of Service**		
Diploma	6	2.8	< 1 year	29	12.5
Bachelor degree	125	54.2	1–3 years	51	22.2
Master degree	80	34.7	3–5 years	58	25
Doctoral degree	20	8.6	> 5 years	93	40.3
Total	231		Total	231	

## Measurement model assessment

The initial step of the PLS-SEM process was analyzing the measurement model. This would ensure the authenticity and credibility of the model. To be considered reliable, Cronbach’s alpha and composite reliability (CR) should be more than 0.70, as Hair et al. (2017) stated. It was determined that a value of less than or equal to 0.50 for the Average Variance Extracted (AVE) was necessary for a convergent validity check. According to Lam (2012), in the event, the AVE is less than 0.5 and the CR is higher than the 0.6 thresholds, the result is accepted. The Cronbach’s alpha values for all the constructs of this study ranged from 0.677 to 0.906, while the CR values were between 0.770 and 0.91. The AVE values range from 0.526 to 0.651. Table 1 displays the results of the reliability and validity measurements for each construct, all of which are within the predetermined threshold.

The Heterotrait Monotrait Ratio (HTMT) was then used to test the model’s discriminant validity (Dijkstra, 2014). According to Hair et al. (2017), the HTMT value must be less than 0.90 for it to be statistically significant. Based on the fact that none of the values in Table 3 were greater than 0.90, it can be deduced that the respondents were aware of the distinction between the constructs; hence, discriminant validity has been established.

**Table 3:** Discriminant validity—Heterotrait-Monotrait ratio (HTMT).

HTMT	MF	PWB
PWB	0.541	
RES	0.609	0.815

## Structural model assessment

Based on Hair et al. (2019), it is advisable for researchers to first and foremost analyze the structural model assessment by measuring the predictive power. R2, Q2, and f2 were measured to achieve this. To be considered significant, the R2 value must be at least 0.25 (Hair et al., 2017). A relevant number for Q2 would be greater than zero. However, for f2, the bare minimum would need to be less than 0.02 for the value to be meaningful. All of the values in this study were within the necessary threshold.

## Verifying the proposed hypotheses

**Figure 2:**
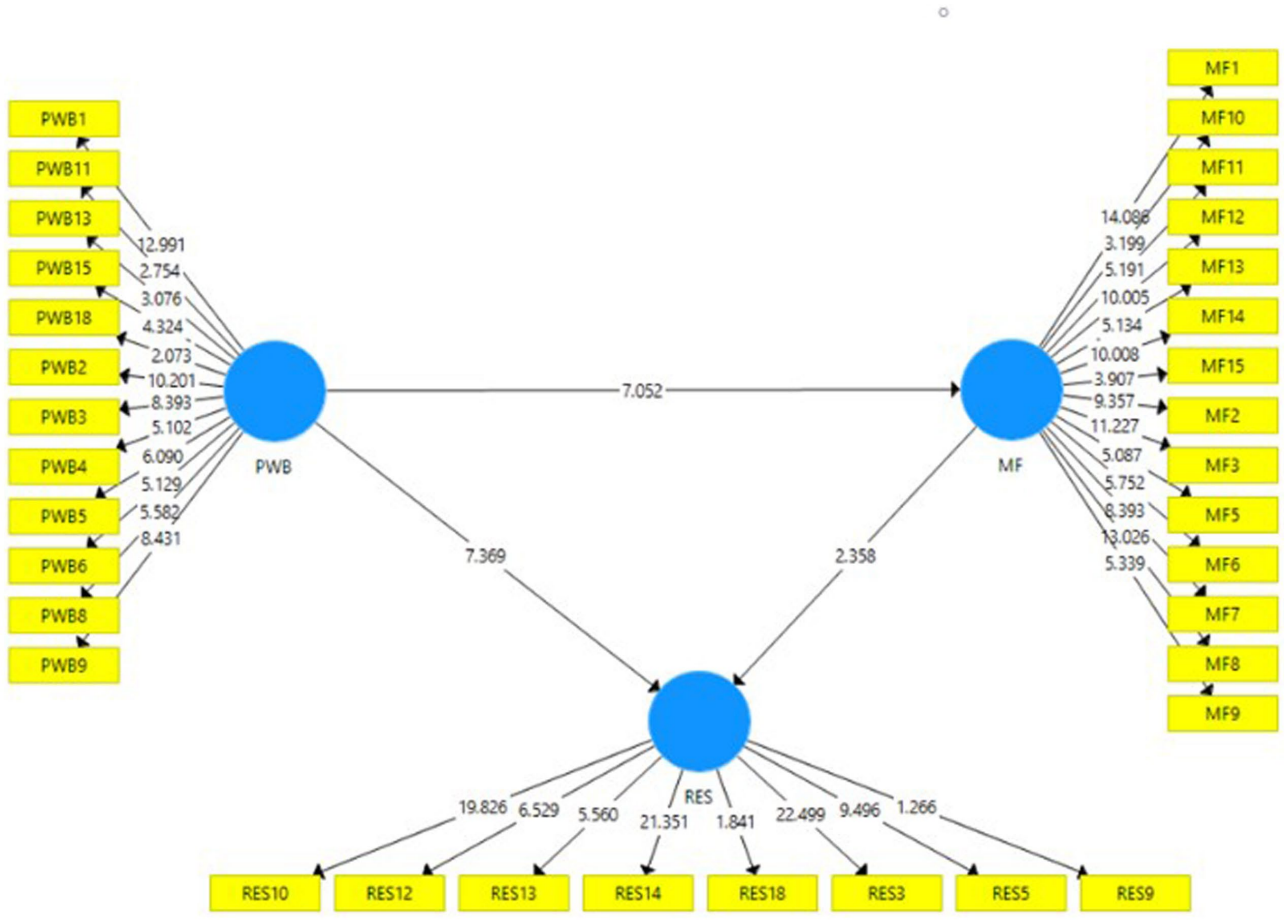
Structural model of the study.

In the process of examining the validity of the hypothesized association between the variables, T-Statistics were employed. The bootstrapping technique examines the construct’s compatibility with the data. By implementing this technique, the real sample size is raised to 5,000, as proposed by Hair et al. (2017). Please refer to Figure 2 and Table 4 for the structural model’s results. Since this was a one-tailed study, a T-statistic of 1.65 or less was considered statistically significant (Hair et al., 2012). When looking at the direct correlations, the data confirmed hypotheses 1, 2, and 3. T-Stats = 2.358; *p* = 0.019 supported the hypothesis that mindfulness practice increases resilience. There were also statistically significant benefits of mindfulness on PWB (T-Statistics = 7.052; *p* = 0.000). Finally, it was discovered that PWB significantly affected resilience (T-Statistics = 7.369; *p* = 0.000). Based on the analysis on the mediation model, it was found that mindfulness mediated the connection between psychological health and fortitude (T-Statistics = 2.098; *p* = 0.036). With that, H4 is also confirmed and considered statistically significant.

**Table 4:** Total effects.

	Original sample	Sample mean	Standard deviation	T statistics	***P***-values
MF - > RES	−0.235	−0.238	0.100	2.358	0.019
PWB - > MF	0.555	0.595	0.079	7.052	0.000
PWB - > RES	−0.612	−0.617	0.083	7.369	0.000
PWB - > MF - > RES	−0.131	−0.141	0.062	2.098	0.036

## Discussion

According to the findings of this study, mindfulness shows a significant relationship in mediating both PWB and the resilience of millennials. The outcomes of the analysis show that a mindful individual reported a high level of PWB and low levels of psychological distress. These findings are consistent with the conclusions drawn from a study by Whitehead et al. (2018), Li and Hasson (2020), and Klainin-Yobas et al. (2021).

When confronted with challenges, those who practice mindfulness have a more optimistic outlook on their lives and are less likely to struggle with mental health issues. This may be one of the reasons why mindfulness preserves the well-being of millennials. It is necessary to conduct additional research on the subfactors of mindfulness engaged in safeguarding the millennial’s well-being. Mindfulness techniques were shown to be the medium that enabled the association between PWB and the capacity of individuals to thrive in an environment fraught with adversity and uncertainty. Therefore, the current study’s findings regarding the relationships between the constructs provide evidence as the basis for the association between mindfulness and millennials’ PWB when faced with adversities.

According to the findings of this research, mindfulness has a strong relationship with resilience. Resilience was discovered to have a positive association with PWB and a negative association with the negative experience of psychological distress. These findings are consistent with those obtained from a variety of earlier studies, both theoretical and empirical, which were carried out in the past (Whitehead et al., 2018; Li and Hasson, 2020; Klainin-Yobas et al., 2021). This supports the idea that teaching millennials to develop their inner strength by practicing mindfulness may be one strategy to help them become more resilient in the face of the many daily stresses they face.

Consequently, this can encourage a more optimistic assessment of their current living situation and lessen the severity of adverse psychological effects. People who practice mindfulness are more likely to have a healthy relationship with the challenges they face, which contributes to the fact that they are better able to cope with the challenges brought on by adversity and uncertainty. Benevene et al. (2020), found that people who regularly practice mindfulness are more likely to use optimistic coping strategies (like positive thinking and maintaining a positive outlook) when facing adversity. They are more likely to view the long-term outcomes of their current situation with optimism (Moss et al., 2017). If today’s millennials take on such a positive and optimistic outlook in the face of hardship, they may see it as a chance to grow and improve (Xi et al., 2015). This may aid their capacity to adapt to adverse conditions, enhancing their chances of survival.

People dealing with difficult circumstances may benefit from practicing mindfulness since it may prevent them from overgeneralizing their precarious life circumstances, which will, in turn, protect PWB. The findings of this study demonstrate that mindfulness substantially impacted the millennials’ PWB and directly affected their resilience level. This supports the hypothesis that mindfulness training can help people become more resilient and less stressed. Mindfulness has been shown to preserve health by lowering stress and increasing resilience (Sünbül and Güneri, 2019). This supports the idea that the processes that lead to greater resilience and the link between mindfulness and PWB are related.

According to Zhu et al. (2019) when considering the effects of extreme adversity that befall an individual from a psychological and social point of view; one of the most important considerations to consider is stress. Based on one similar study undertaken by Li (2021) based on this finding, a person who manages successfully bounces back from adversity is more likely to have an optimistic outlook on the world. By increasing one’s resiliency in the face of adversity, mindfulness may make it possible for a person to have a more optimistic subjective view of the challenging situation (Li, 2021). This is particularly relevant for millennials who live in a world that is full of challenges. With that, the mediation role of mindfulness as an intervention factor will improve PWB and increase resilience, leading to a reduced level of negative mental issues. This firmly explains the relationship between the constructs whereby mindfulness has significant positive influences on the PWB and increased level of resilience of the millennials.

## Conclusion

Mindfulness techniques that are practiced regularly appear to be an essential internal resource that can assist millennials in adjusting to the life circumstances they are currently facing. A mindful person is less prone to experience adverse PWB due to the influence of their surroundings. This is likely, due to the ability to regulate their focus. Mindful individuals are more successful at adapting to the adverse situation they experience. With that, their thoughts and emotions are observed and regulated. Thus, they are less adversely affected by negative influences. As a result, the level of the PWB of more mindful individuals will experience less likelihood of being threatened by uncertainty and environmental-related adversities. Based on the result presented in this study, it is possible to conclude that engaging in regular mindfulness activities helps preserve the PWB of the millennials. It is essential that the many tools and programs that make it easier for millennials to begin mindfulness practices be available. This will allow for more significant improvements in the overall resilience that millennials possess when confronted with future challenges. Accordingly, this study can offer insights into how to deal with such difficulties and disturbances through appropriate tactics and can help create a relaxing environment where working millennials can work happily and perform at a higher level. Additionally, businesses can provide mindfulness exercises to millennial employees and design a mindfulness learning environment that will increase employee engagement and motivation.

## Research limitation

More qualitative and long-term studies could be conducted in the future to better understand how mindfulness can be put in place to support the level of resilience of working millennials. By including more participants from other nationalities in the sample size for subsequent research, the results might be more broadly applicable.

## Suggested future research

In this current study’s similar methodology, that mainly focuses on the working millennials with the three variables (PWB, Resilience, and Mindfulness) and has neglected the view of other possible categories of the population. With that, for further or future researchers, it can be recommended to consider conducting comparable studies that incorporate the viewpoints of the other categories of populations. In another aspect of this study, PWB is the emphasis that has received more attention in this study’s variables as compared to mindfulness and resilience of working millennials. Therefore, as another suggestion, researchers are encouraged to investigate these two variables (mindfulness and resilience) with other identified factors by other similar research. The factors such as interpersonal communication behaviors, self-efficacy, happiness, and optimism could play an important part in providing further understanding of the millennials.

Another suggestion to further explore the study of PWB, mindfulness, and resilience, is to take into consideration the influence of demographic elements, such as age, working experience, academic level, income level, gender, and others. This current study has neglected and did not take into consideration the demographic aspects of the participant of this study, as can be seen from reading the connected studies in this study area. These are all the elements that are lacking which can be a potential element for consideration for future studies and it is evident that this field of study is still in its early stages and requires additional research.

## Data availability statement

The raw data supporting the conclusions of this article will be made available by the authors, without undue reservation.

## Author contributions

VO designed the study. VO and NP collected the data. VO and AS analyzed and interpreted the data. VO and NP drafted the manuscript. VO and AS revised the manuscript critically for important intellectual content. All authors agreed to be accountable. All authors contributed to the article and approved the submitted version.

## Funding

This work was supported by the Ministry of Higher Education, Malaysia under Fundamental Research Grant Scheme (Grant number: FRGS/1/2020/SS01/MMU/03/4).

## Conflict of interest

The authors declare that the research was conducted in the absence of any commercial or financial relationships that could be construed as a potential conflict of interest.

## Publisher’s note

All claims expressed in this article are solely those of the authors and do not necessarily represent those of their affiliated organizations, or those of the publisher, the editors and the reviewers. Any product that may be evaluated in this article, or claim that may be made by its manufacturer, is not guaranteed or endorsed by the publisher.
